# Relationship between Deck Level, Body Surface Temperature and Carcass Damages in Italian Heavy Pigs after Short Journeys at Different Unloading Environmental Conditions

**DOI:** 10.3390/ani7020010

**Published:** 2017-02-10

**Authors:** Agnese Arduini, Veronica Redaelli, Fabio Luzi, Stefania Dall’Olio, Vincenzo Pace, Leonardo Nanni Costa

**Affiliations:** 1 Department of Agricultural and Food Sciences, School of Agriculture and Veterinary Medicine, University of Bologna, Via Fanin 50, Bologna 40127, Italy; vereda@tin.it (V.R.); stefania.dallolio@unibo.it (S.D.); leonardo.nannicosta@unibo.it (L.N.C.); 2 Department of Veterinary Science and Public Health, Faculty of Veterinary Science, University of Milan, Via Celoria 10, Milan 20133, Italy; fabio.luzi@unimi.it; 3 OPAS, Pig Farmer Association, Strada Ghisiolo 57, San Giorgio, Mantua 46030, Italy; vincenzo.opas@coopgsp.it

**Keywords:** pigs, transport, distance, body surface temperature, carcass, damage

## Abstract

**Simple Summary:**

Transport duration and thermal conditions can negatively affect pig welfare and carcass quality. The effects of short journeys (30 min) in different thermal-humidity conditions on the body surface temperature of live heavy pigs and carcass skin damage were examined. Body temperature increased with increasing Temperature Humidity Index (THI) class. The highest and lowest body surface temperatures were found in pigs located on the middle and upper decks, respectively. THI class significantly affected skin damage scores, which increased with increasing THI class. Even at relatively low temperatures and THI, the results of this study suggested the need to increase the control of environmental conditions in the truck during short-distance transport of pigs, in order to improve welfare and reduce loss of carcass value.

**Abstract:**

In order to evaluate the relationships between deck level, body surface temperature and carcass damages after a short journey (30 min), 10 deliveries of Italian heavy pigs, including a total of 1400 animals from one farm, were examined. Within 5 min after the arrival at the abattoir, the vehicles were unloaded. Environmental temperature and relative humidity were recorded and a Temperature Humidity Index (THI) was calculated. After unloading, maximum temperatures of dorsal and ocular regions were measured by a thermal camera on groups of pigs from each of the unloaded decks. After dehairing, quarters and whole carcasses were evaluated subjectively by a trained operator for skin damage using a four-point scale. On the basis of THI at unloading, deliveries were grouped into three classes. Data of body surface temperature and skin damage score were analysed in a model including THI class, deck level and their interaction. Regardless of pig location in the truck, the maximum temperature of the dorsal and ocular regions increased with increasing THI class. Within each THI class, the highest and lowest body surface temperatures were found in pigs located on the middle and upper decks, respectively. Only THI class was found to affect the skin damage score (*p* < 0.05), which increased on quarters and whole carcasses with increasing THI class. The results of this study on short-distance transport of Italian heavy pigs highlighted the need to control and ameliorate the environmental conditions in the trucks, even at relatively low temperature and THI, in order to improve welfare and reduce loss of carcass value.

## 1. Introduction

The transport to the slaughterhouse is considered an important stressor for slaughter pigs, which may have deleterious effects on health and welfare, as well as carcass and pork quality [1]. Several studies have been conducted to assess the influence of factors connected to the transport, including loading density, distance and duration of journey, handling treatment, trailer design, loading method, environmental conditions and internal microclimate [2]. In particular, the thermal environment affects physiological and behavioural responses of transported pigs [3]. Warm environmental conditions are associated with a higher risk of fatigue, open-mouth breathing and death in-transit or at arrival [4]. Moreover, both cold and heat stress affect muscle glycogen stores leading to an increased incidence of pale soft exudative (PSE) or dark firm and dry meat defects [5]. Environmental conditions during transport, that also affect skin blemishes and meat quality, include the position of the animal inside the vehicle [6,7]. Pigs located in the front and rear compartments or in the upper and lower decks showed an increased number of carcass skin bruises and a reduced pork quality [8]. Despite several studies on the influence of the environmental conditions during long-distance transports on pig welfare and carcass quality, few studies have investigated these effects during short-distance transports. Short-distance transports are very common in many European regions with high concentrations of piggeries and slaughter plants [9]. Gajana et al. [10], Guardià et al. [11] and Perez et al. [12] observed a higher incidence of PSE meat in pigs that had been transported a short duration compared to pigs transported longer distances. Barton Gade and Christensen [13] reported an increased risk of skin damage during short-distance transports (2–3 h). Gispert et al. [14] also found higher skin damage scores in short-distance transports (<2 h) compared to long-distance transports (>2 h). Conversely, Mota-Rojas et al. [15] found that the frequency of bruised carcasses increased with journey duration. Hence, there is a need to increase the knowledge on the relationships between short-distance transport and skin damages. An important topic in animal welfare study concerns the measure of stress avoiding invasive methods. Infrared thermography may be considered a reliable and non-invasive tool to evaluate the stress impact of different management practices at the farm and at slaughter [16]. The aim of this study was to evaluate the relationship between the deck level, dorsal and ocular region surface temperature and carcass damage after a short duration transport of Italian heavy pigs destined for the dry-cured industry.

## 2. Experimental Section

### 2.1. Data Collection

This study was carried out on data collected from 1400 crossbred (Duroc × (Landrace × Large White)) Italian heavy pigs (live weights average ± s.d.: 171.1 ± 6.1 kg) provided in 10 batches supplied by the same farm on 10 different days, randomly chosen between January and June 2014. Each batch consisted of 140 pigs 9 months old, according to the denomination of protected origin dry-cured ham Parma Consortium [17]. The plant was located in Northern Italy with a chain speed of 280 pigs/h.

### 2.2. Pre-Slaughter Conditions and Slaughterhouse

**Figure 1:**
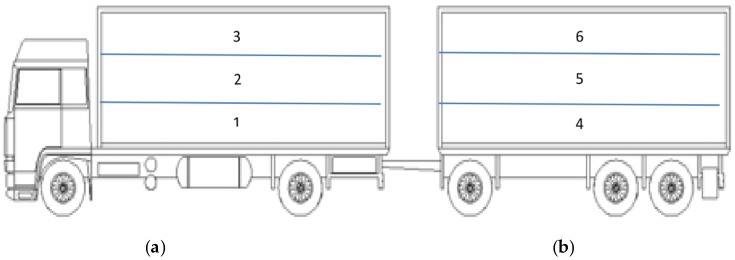
Schematic drawing of the truck with hydraulic decks used for pig transportation. The lorry was always unloaded before the trailer and decks were unloaded sequentially, starting with the lower deck. (**a**) LORRY; (**b**) TRAILER.

The trucks were stocked according to European livestock transport rule EC Regulation No. 1/2005 [18] with an available surface of about 0.73 m^2^/pig. The pigs were transported on rural and secondary roads with a journey time of 31 ± 5 min at an average speed of 30 km/h. The transport was always carried out using a truck from Carrozzeria Pezzaioli (Montichiari, Italy), composed of a main lorry and a trailer, each one equipped with three hydraulic decks (Figure 1) containing 23–24 pigs/deck. Lorries and trailers had both natural and mechanical ventilation systems, with automatic fans placed on the left side of both compartments. The fans, nine in each truck, were 225 mm in diameter with a 11,700 m^2^/h flow. Pigs were off feed for 12 h before transport. Loading was carried out at around 06:00 a.m. by three farm operators, avoiding mixing between unfamiliar pens. The farm operators went out to one pen at a time to drive pigs toward the loading platform, using a plastic stick. The loading procedures lasted approximately 45 min. A mobile ramp (length 6.0 m, width 0.7 m, with 1.0 m solid side walls and adjustable height) was used to load pigs into the truck. Within 5 min after the arrival at the abattoir, the pigs were unloaded using a fixed platform (length 9.3 m, width 2.7 m, 1.0 m solid side walls) that was height-adjusted to the level of the lower deck by the lairage manager and the driver. Each deck was unloaded within 2 min and the complete unloading procedures lasted approximately 12 min. The lorry was always unloaded before the trailer and the decks were unloaded sequentially, starting with the lower deck, as shown in Figure 1.

During the unloading of each deck, environmental temperature (°C) and relative humidity (%) were recorded using a Gemini Tinytag Ultra 4500 Thermal Sensor (Gemini Data Logger Ltd., Chichester, West Sussex, UK) located on a windowsill close to the platform, at the entrance of the resting pens. A Temperature Humidity Index (THI) was then calculated according to NRC [19] using the formula: THI = {(1.8 × T + 32) – (0.55 − (0.0055 × RH))} × {(1.8 × (1.8 × T + 32)) − 26}, in which T is the temperature and RH is the relative humidity. Order and date of deliveries and temperature (T), relative humidity (RH) and THI measured at each delivery during unloading are presented in Table 1.

At the plant, pigs were driven with plastic sticks or rubber boards to resting pens and they were allowed to rest for 20 to 30 min. During this period, pigs were not mixed with unfamiliar animals. After the rest, the pigs were showered and driven through a single passageway to the stunning cage. Stunning was manually done by electrical tongs (head only; 170 V, 1.3 A). Carcasses were horizontally exsanguinated for 3 min, then hanged for 10 min before being immersed in a scalding tank for dehairing at 62 °C for 10 min. After dehairing, skin damages were subjectively assessed by the same trained technician, using a four-point scale (1 = none to 4 = severe) based on the scale developed by the Danish Meat Research Institute (DMRI) [20]. The DMRI scale was used to score all skin lesions on the front (head included), middle and hind quarters of each carcass. Moreover, a skin damage score for the whole carcass was calculated using the highest score assigned to each quarter [20]. The carcasses were then eviscerated, split, hot-boned and sectioned in primal cuts.

### 2.3. Thermal Imaging

**Figure 2:**
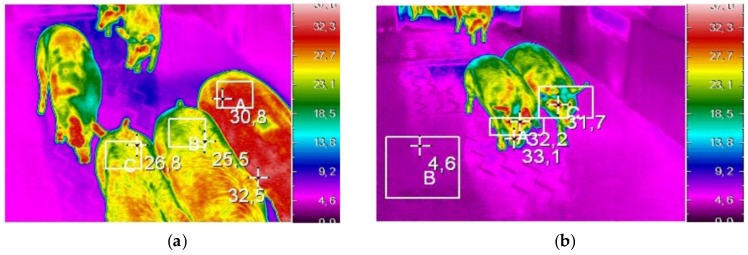
Examples of thermal images of the dorsal (**a**) and ocular (**b**) regions recorded during the unloading of Italian heavy pigs at the slaughterhouse after transport of approximately 30 min under commercial conditions. The white rectangles indicate the areas in which the maximum temperatures were recorded.

During the unloading of each deck, as the pigs were driven along the platform, the maximum surface temperatures were recorded on the dorsal and ocular regions using an Avio thermoGear Nec G120 EX thermal camera (Nippon Avionics Co., Ltd., Tokyo, Japan). Several studies have found that these regions show changes in maximum surface temperatures in response to acute stress and variation in environmental conditions [21,22]. The camera was calibrated manually before to carry out measurements on pigs from each delivery. The camera was located at the entrance of the lairage pens at about 3 m from the unloading point and at a height of 1.80 m. The skin emissivity was manually set in the camera at 0.96 before each unloading. Thermal images were downloaded to a computer and examined using NEC InfRec Analyzer and Grayess IRT Analyzer software (Nippon Avionics Co., Ltd., Tokyo, Japan). A total of 7000 readable thermal images including 1222 pigs were analysed to determine the maximum surface temperatures within the dorsal and ocular regions. An example of the examined thermal images is shown in Figure 2.

### 2.4. Statistical Analysis

For the statistical analyses, a classification of deliveries on the basis of THI was done using the CLUSTER procedure of SAS (SAS Institute, Cary, NC, USA) [23]. Using a dendogram analysis, three clusters were identified. The FASTCLUS procedure of SAS [23] was used in order to classify the deliveries into one of the three clusters previously defined. In this procedure, observations that are very close to each other are assigned to the same cluster by an algorithm for minimizing the sum of squared distances from the cluster means [23]. Deliveries classification and means and standard deviations of environmental temperature, relative humidity and THI for each THI class are reported in Table 2. All data were tested for normality by using PROC UNIVARIATE of SAS [23]. A preliminary analysis was conducted to assess differences between the lorry and trailer. No significant differences (*p* > 0.05) were found, therefore this source of variation was not included in subsequent statistical analyses. Data of maximum surface temperatures were analysed using the GLIMMIX procedure of SAS using a model including THI class, deck level and their interaction. GLIMMIX was also used to analyse the effects of the same sources of variation on skin damage scores recorded on each quarter separately as well as on the whole carcass. Because these data approximated a Poisson distribution, the GLIMMIX procedure’s POISSON option was used. The ILINK option was used to back-transform least squares means of the skin damage score. The differences in least squares means were evaluated using Tukey-Kramer’s test. The level for statistical significance used was *p* < 0.05 in all analyses.

## 3. Results and Discussion

THI class, deck level and their interaction significantly influenced the maximum surface temperatures of the dorsal region (*p* < 0.05). The interaction effect on these temperatures is shown in Table 3. Within each deck, the surface temperatures increased significantly (*p* < 0.05) with increasing THI class. This result agrees with findings of several studies reviewed by Soerensen and Pedersen [24] who pointed out that body superficial temperature increased at high ambient temperature and decreased at low ambient temperatures. A significant effect of deck level was observed in the first THI class only (*p* < 0.05) where the mean value recorded on pigs located on the upper deck of the trailer (deck 6) was significantly lower compared to the middle and lower decks of the whole truck. In the second THI class, there was a tendency in pigs located on the upper deck of the trailer (deck 6) to show the lowest surface temperature while in the third class, the lowest mean values were recorded on pigs located in the lower deck of the trailer (decks 4). Probably, due to a short-distance transport time, the variation of ambient temperature and internal air flow between decks within THI classes had a weak impact on the thermoregulation of pigs. 

In terms of the maximum surface temperature of the ocular region, an interaction between THI class and deck level was again observed (Table 4). As in the dorsal region, the maximum surface temperatures of the ocular area increased with increasing THI class. A significant effect of deck (*p* < 0.05) was observed in the first and second THI classes only. In the first THI class, the highest mean values of ocular surface temperature were recorded on pigs located on the middle deck of the lorry (deck 2) and in the lower deck of the trailer (deck 4), whereas the lowest values were found on subjects located on the upper deck (decks 3). In the second THI class, the lowest mean values were found in pigs located on the upper deck (deck 6), whereas the highest values were observed in pigs transported in the middle decks of trailers (deck 5). A tendency towards a higher temperature on lorry decks 2 and 3 was observed in the third THI class.

In general, in the present study, the maximum surface temperatures recorded in the ocular region were slightly lower compared to those recorded in the same region on pigs restrained in cages [21,24]. This is probably due to differences in the distance [25] between the thermal camera and the pigs at unloading compared to pigs restrained into cages.

For the skin damages, only the THI class had a significant effect (*p* < 0.05) on scores recorded both in individual quarters and the whole carcass (Table 5). Even at relatively low temperatures and THI, the general pattern observed was an increase in the skin damage score in all quarters with increasing THI class. In the hind and front quarters, as well as in the whole carcass, the mean score of the third THI class was significantly higher than those of the first and second classes (*p* < 0.05), which were not significantly different. In the middle quarter, the mean scores of the third THI class were also higher than the other two classes, and the middle THI class was significantly higher than the lowest THI class (*p* < 0.05). Probably, warmer environments resulted in pigs being more active and prone to hits against loading and unloading facilities. These results agree with Dalla Costa et al. [5] and Eldridge and Winfield [26] who found that environmental factors, such as temperature and humidity, affect the incidence of skin bruises. In a recent study on the effects of season and location inside the truck on pig behaviour, Torrey et al. [27] observed more slips, falls, overlaps and backward at unloading in summer than in winter. Also in cattle, skin damage scores were found to be higher in warmer environments [28]. In particular, Mpakama [29] showed that higher temperatures, especially in summer, increased the risk of skin bruises on arrival at the plant. 

It could be argued that maintaining pigs at low space and high THI in a stationary vehicle at the slaughterhouse can lead to aggression even among well-acquainted animals. It is unlikely that this occurrence can explain the effect of THI class on the increase of skin damage because of the short time spent to complete unloading procedures and the behaviour of familiar heavy pigs. Martelli et al. [30] found very low incidence of aggressive interactions in well-acquainted Italian heavy pigs kept at different light intensity. Moreover, during all deliveries, there was no evidence of aggressions among pigs in the stationary vehicles. 

Deck level did not significantly affect skin damage scores (data not shown). A tendency towards higher skin damage scores was found in pigs transported on the trailer compared to those located on the lorry. This tendency could be explained by the fact that the trailer is subjected to more vibrations and movements compared to the lorry. 

## 4. Conclusions

The results of this study on short-distance transport of Italian heavy pigs confirm that different environmental conditions, even when experienced during a limited time, affect surface body temperatures and carcass skin damages. The location of the pigs in the vehicle interacts with environmental conditions in their effects on surface body temperatures, but not on carcass damage scores. Thermal and humidity conditions played an important role on the skin damage score which appeared to increase with increasing THI. If the effect of THI increase on the risk of skin damages is confirmed by further studies, then the control of environmental conditions during short-distance transport of pigs will be one of the main concerns from a welfare perspective. However, more research is needed to determine the effect of short-time transport on the stress and carcass damages in heavy pigs during different environmental conditions, such as extreme THI values.

## Figures and Tables

**Table 1:** Identification (ID) and date of deliveries and outdoor temperature (T), relative humidity (RH) and Temperature Humidity Index (THI) recorded at unloading. The sensor was located close to the platform crossed by the pigs to reach the resting area.

Delivery ID	Date (yy/mm/dd) *	T (°C)	RH (%)	THI
1	14/01/28	5.6	91.2	42.9
2	14/02/11	5.8	94.1	42.9
3	14/03/11	9.5	70.4	50.6
4	14/03/18	7.0	79.0	46.1
5	14/03/25	4.0	72.0	42.1
6	14/04/01	14.7	66.9	58.4
7	14/04/15	14.2	77.8	57.6
8	14/05/20	19.6	69.0	65.7
9	14/05/27	17.6	80.9	63.1
10	14/06/10	22.0	51.0	67.9

* yy/mm/dd: year/month/day.

**Table 2:** Distribution based on the Temperature Humidity Index (THI) of the ten deliveries of Italian heavy pigs transported approximately for 30 min under commercial conditions. For each class of THI, identification (ID) of the deliveries, number of pigs, mean and standard deviation (s.d.) of outdoor temperature (T), relative humidity (RH) and THI at unloading are shown.

THI Class *	Deliveries ID	Pigs	T (°C)		RH (%)		THI	
		No	Mean	s.d.	Mean	s.d.	Mean	s.d.
1	1, 2, 4, 5	560	5.6	1.1	76.6	2.8	43.5	1.5
2	3, 6, 7	420	12.8	2.4	71.7	4.6	55.5	3.5
3	8, 9, 10	420	19.7	1.8	67.0	12.3	65.6	2.0

* THI classes were obtained by the CLUSTER procedure on the basis of THI values and the deliveries were allocated into the classes by the FASTCLUS procedure of SAS [23].

**Table 3:** Effect of the interaction between the class of the Temperature Humidity Index (THI) and deck level on the maximum surface temperature (°C) recorded at unloading by a thermal camera on the dorsal region of Italian heavy pigs transported approximately for 30 min under commercial conditions (least squares means).

THI Class *	Deck Level						SE
	1	2	3	4	5	6	
1	28.6 **^x ab^**	29.1 **^x ab^**	27.8 **^x ab^**	28.7 **^x ab^**	29.5 **^x a^**	27.3 **^x b^**	0.26
2	31.1 **^y^**	31.6 **^y^**	31.2 **^y^**	30.9 **^y^**	31.1 **^y^**	30.5 **^y^**	0.30
3	34.4 **^z^**	35.0 **^z^**	34.2 **^z^**	33.7 **^z^**	34.3 **^z^**	34.1 **^z^**	0.34

**^a^**^, **b**^: different superscript letters in the same row are different at *p* < 0.05; **^x^**^, **y**, **z**^: different superscript letters in the same column are different at *p* < 0.05. SE: standard error. * THI classes included the following deliveries (ID): 1, 2, 4 and 5 in class 1; 3, 6 and 7 in class 2; 8, 9 and 10 in class 3.

**Table 4:** Effect of the interaction between the class of the Temperature Humidity Index (THI) and deck level on the maximum surface temperature (°C) recorded at unloading by a thermal camera on the ocular region of Italian heavy pigs transported approximately for 30 min under commercial conditions (least square means).

THI Class *	Deck Level						SE
	1	2	3	4	5	6	
1	32.8 **^x ab^**	33.2 **^x a^**	32.0 **^x b^**	33.2 **^x a^**	33.0 **^x ab^**	32.4 **^x ab^**	0.60
2	33.7 **^y ab^**	34.2 **^y ab^**	33.7 **^y ab^**	33.8 **^y ab^**	34.5 **^y a^**	33.5 **^y b^**	0.74
3	35.1 **^z^**	35.4 **^z^**	35.3 **^z^**	35.1 **^z^**	35.1 **^z^**	35.1 **^z^**	0.87

**^a^**^, **b**^: different superscript letters in the same row are different at *p* < 0.05; **^x^**^, **y**, **z**^: different superscript letters in the same column are different at *p* < 0.05. SE: standard error. * In THI classes, the following deliveries (ID) were included: 1, 2, 4 and 5 in class 1; 3, 6 and 7 in class 2; 8, 9 and 10 in class 3.

**Table 5:** Effect of the Temperature Humidity Index (THI) class on the skin damage score **^(1)^** of single quarters and whole carcass of Italian heavy pigs (least squares means).

Skin Damage Score	THI Class			SE
	1	2	3	
Single quarters:				
-hind	2.07 **^b^**	2.26 **^b^**	2.59 **^a^**	0.07
-middle	2.56 **^c^**	2.83 **^b^**	3.50 **^a^**	0.08
-front	2.10 **^b^**	2.14 **^b^**	2.58 **^a^**	0.07
Whole carcass	2.75 **^b^**	2.98 **^b^**	3.58 **^a^**	0.08

**^(1)^** four-point scale: 1 = none to 4 = severe; **^a^**^, **b**, **c**^: different superscript letters in the same row are different at *p* < 0.05. SE: standard error.
